# Fabrication of a Lactate-Specific Molecularly Imprinted Polymer toward Disease Detection

**DOI:** 10.1021/acsomega.2c08127

**Published:** 2023-02-21

**Authors:** Yasemin L. Mustafa, Hannah S. Leese

**Affiliations:** † Materials for Health Lab, Department of Chemical Engineering, University of Bath, Bath BA2 7AY, U.K.; ‡ Centre for Biosensors, Bioelectronics and Biodevices, University of Bath, Bath BA2 7AY, U.K.

## Abstract

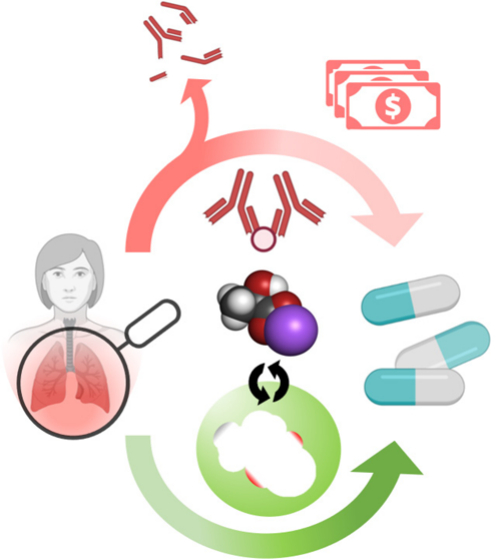

The development of sensitive and selective robust sensor materials for targeted biomarker detection aims to contribute to self-health monitoring and management. Molecularly imprinted polymeric (MIP) materials can perform as biomimetic recognition elements via tailored routes of synthesis for specific target analyte extraction and/or detection. In this work, a sensitive- and selective-lactate MIP has been developed utilizing methacrylic acid and ethylene glycol dimethacrylate as the functional monomer and cross-linker, respectively. The sensitivity of the as-synthesized imprinted species was evaluated by determining the target analyte retention, imprinting factor, and selectivity adsorption of up to 63.5%, 6.86, and 0.82, respectively. MIP selectivity elucidated the imprinting mechanism between the functional monomers and target analyte lactate, further experimentally evidenced by using structurally competitive analytes malic acid and sodium 2-hydroxybutyrate, where retentions of 22.6 and 25.2%, respectively, were observed. Understanding the specific intermolecular mechanisms of both the template analyte and structural interferents with the MIP enables experimentalists to make informed decisions regarding monomer-target and porogen selections and possible sites of interaction for improved molecular imprinting. This imprinting system highlights the potential to be further developed into artificial receptor sensor materials for the detection of disease.

## Introduction

Infections, a prominent cause of hospital mortality, often lead to dynamic diseases like sepsis. Sepsis is described as *a life-threatening organ dysfunction caused by an infection-induced dysregulated host response*, according to the Surviving Sepsis Campaign guidelines.^1^ Early diagnosis of sepsis is paramount for saving lives and preventing long-term health effects such as permanent organ dysfunction, cognitive impairment, and/or loss of appendages.^2^ Circulating blood lactate can be utilized as a marker for systemic tissue hypoperfusion (reduced blood flow), reflective of cellular dysfunction in septic patients.^3^ Antinone et al. credited the linear range of healthy venous and arterial blood lactate between 0.5 and 2.2 mM and 0.5 and 1.6 mM, respectively.^4^ An elevated blood lactate concentration (>2 mM) is recognized as hyperlactatemia, and in extreme cases (>5 mM) this can develop into lactic acidosis.^5^ Deviations in lactate concentrations are attributed to the disturbance of the lactate metabolism, often a consequence of infection.^6^ Therefore, monitoring biomarkers, present in body fluids or tissues, such as lactate, can perform as fundamental indicators for understanding the biological mechanisms occurring within the human body during periods of disease.

Currently, in the clinic, concentration levels of lactate are monitored via blood gas analyzers, which sample arterial blood. Equipment of this caliber is expensive, requires training to operate, uses invasive collection methods, and needs large sample volumes and long sample-processing times, making them unsuitable for diagnosis/treatment, particularly at the point-of-care (POC).^7^ At present, amperometric meters have been developed for lactate biomarker analysis sampled from blood serum, sweat, and brain and peripheral tissues.^8−10^ These devices are reliant on enzyme species, which can often suffer from poor physicochemical stability (e.g., pH and temperature changes), resulting in degradation and eventually denaturation, whereby enzyme characteristics cannot be recovered. Moreover, the inadequate shelf life of these species can have detrimental effects on the long-term sensing capabilities of these devices.

An alternative approach could be the incorporation of molecularly imprinted polymers (MIPs) as the sensor component of future POC devices. To achieve this incorporation, sensitive and selective MIPs must be developed. MIPs have the ability to be robust and environmentally stable recognition materials, which imitate the behavior of naturally occurring biological receptors, to detect small biomolecules and antibodies.^11−13^ The versatile recognition ability of MIPs extends to both chemical and biological molecules, including nucleotide derivatives, amino acids, pollutants, and proteins.^14−17^ Moreover, sensor elements using biological species face significant drawbacks with respect to temperature sensitivity, functionality, and shelf life. Custom-made synthetic MIP species can overcome these issues due to the excellent physicochemical characteristics of MIP technologies.^18,19^ Currently, routine means to measure lactate levels continuously in a noninvasive manner are clinically deficient. Although MIPs designed for the detection of the lactate biomarker can be found within the current literature,^20−24^ there is a scarce presence of lactate MIPs formed via free-radical bulk polymerization available for therapeutic purposes. Therefore, this investigation seeks to understand the mechanistic interactions between the synthesized polymeric system and the imprinted target analyte lactate that with further developments could facilitate continuous lactate monitoring. There is still a demand for an accurate, rapid, reliable, cost-effective, sensitive, and selective lactate sensor device at POC, which can inform the prescription of specific antibiotics only when necessary.

In this work, an MIP-sensitive and selective toward lactate was synthesized and characterized in a component ratio of 1:6:30 of template (sodium lactate), functional monomer (methacrylic acid, MAA), and cross-linker (ethylene glycol dimethacrylate, EGDMA), respectively. This component ratio proved to be optimal for the target molecule cortisol in our previous work.^25^ The main sites of interaction between the lactate–monomer complex have been investigated, and evaluation of the binding capabilities of this widely used polymeric system aims to strengthen the understanding of the template–monomer interactions. In this study, a linear range of 0.1–1.7 mM was selected to readily identify deviations from recognized “normal” lactate levels. This range was selected due to levels reported in the literature; for example, Madden et al. presented an average interstitial fluid lactate concentration of 1.17 ± 0.23 mM as the norm for 15 healthy volunteers.^26^ More recently, Madden et al. highlighted a healthy salivary lactate of approximately 0.11–0.56 mM.^27^ Additionally, Bakker and Auden et al. attributed a concentration of 1.3 ± 0.07 (*n* = 32) and 1.25 ± 0.05 (*n* = 53) mM as a healthy level of blood lactate for men and women, respectively.^28,29^ Thus, to satisfy the overall aims of this study, 1.7 mM was selected as the upper (cutoff) concentration.^5,30^ Furthermore, to assess the selective capabilities of the MIP, malic acid and sodium 2-hydroxybutyrate were tested as competitive interferants, where the retention of each was measured. These molecules were selected due to their structural resemblance to that of the target (lactate) (see Figure S1i,ii).

## Results and Discussion

### Synthesis and Characterization of MIP and NIP

**Figure 1:**
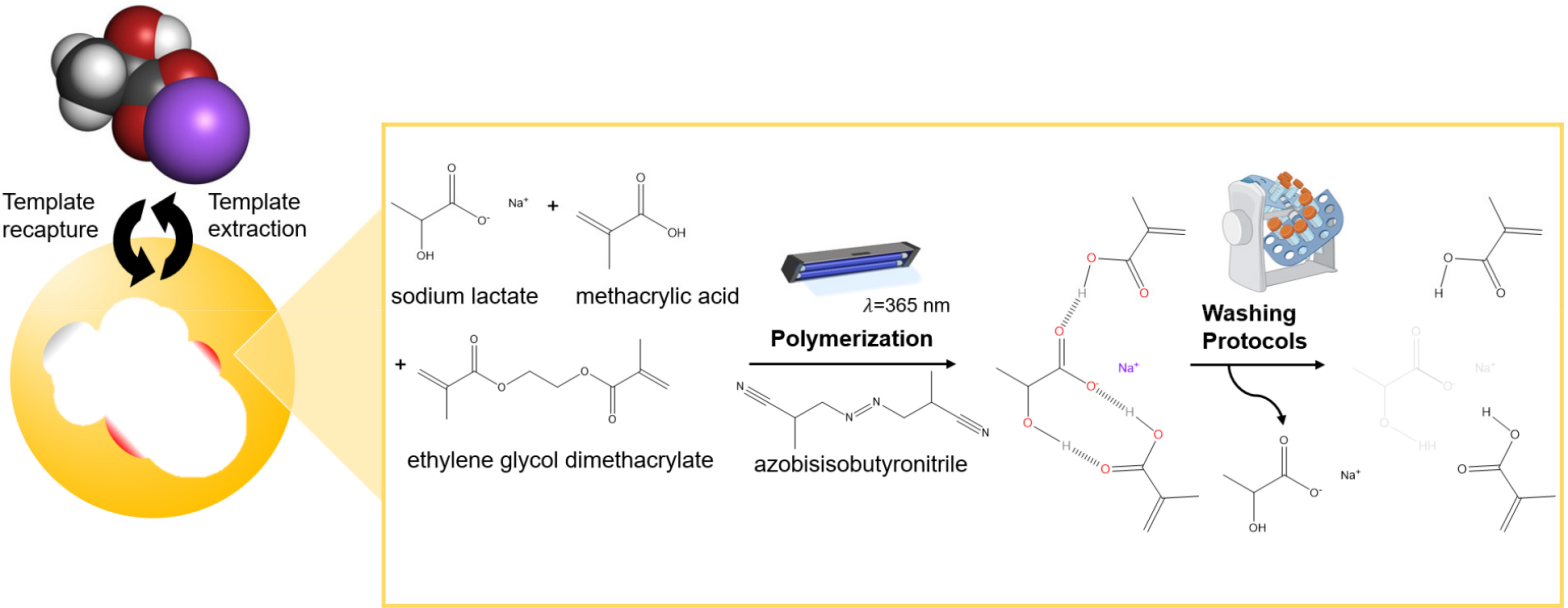
Molecular imprinting of lactate is described by the dissolution of the template (sodium lactate) in the presence of the functional monomer (methacrylic acid) and cross-linker (ethylene glycol dimethacrylate). Polymerization occurs via UV initiation, utilizing the self-assembly approach. The predominant sites of interaction between sodium lactate and methacrylic acid have been highlighted, revealing installed lactate cavities receptive to the target analyte subsequent to washing protocols involving phosphate-buffered saline.

Molecular imprinting via free-radical polymerization is often initiated by dissolution of the target analyte, functional monomer(s), cross-linker, and initiator in an appropriate solvent (Figure 1).^31,32^ Functional monomer(s) are specifically selected for their interaction with the template since their ability to form a stable template-monomer complex is critical for the recognition capability of the imprinted material.^33^ The spatial arrangement of monomer(s) around the template molecule is secured via polymerization aided by a cross-linking agent forming a 3D polymeric network. The formation of interactions (either covalent or noncovalent) between the template and monomer(s) within the polymer matrix are responsible for enabling molecular recognition.^34,35^ Removal of the template results in the formation of binding sites complementary in shape, size, and chemical functionality to that of the template (in this case lactate). Hence, following synthesis, when the MIPs are exposed to the target analyte again, a binding event occurs, which can be measured (the designed recapturing protocol is outlined in Figure S2).

Recent work in developing molecularly imprinting devices for the detection of cortisol, outlined by Parlak et al.,^36^ and our recent work in molecular dynamics simulations with the MAA imprinted system^25^ found that varying the component ratio of the prepolymerization mixture had a significant effect on imprinting quality. Taking the optimal component ratio of the cortisol polymeric system informed this current work, that the key molecular interactions would be between the carboxylic acid functional group of MAA and the alcohol and carboxylate functional groups of lactate. The synthesis of the lactate–MIP followed a radical polymerization (λ = 365 nm) and was mechanically ground and sieved to produce fine powders (ca. 7 μm). The template molecule was extracted by successive MIP powder washings in phosphate-buffered saline (PBS) (Figure S3i). The extraction solvent, PBS, was effective in dissolving the template molecule that was stabilized within the 3D polymeric network during synthesis.^37^ A nonimprinted polymer (NIP) analogue was synthesized under identical conditions (including washings) in the absence of lactate (Figure S3ii) to determine nonspecific adsorption.

Differential scanning calorimetry (DSC) and thermogravimetric analysis (TGA) were performed to characterize the thermal properties of the imprinted species. The DSC and TGA traces of washed MIP and NIP particles are shown in Figures S4i,ii and S5i,ii, respectively. Associated glass transition (*T*_g_) and thermal decomposition (*T*_d_) temperatures are highlighted in Table S1. MIP and NIP species are shown to reach a maximum rate of mass loss at 400 and 405 °C, respectively, indicative of maintained MIP thermal stability.^38^ The NIP appears to show a slightly larger *T*_g_, signifying a greater degree of cross-linking due to the absence of the target analyte. The observed temperature differences are not particularly significant; thus, the lactate template can be installed within the polymer matrix without drastically modifying the thermal characteristics of the material.

**Figure 2:**
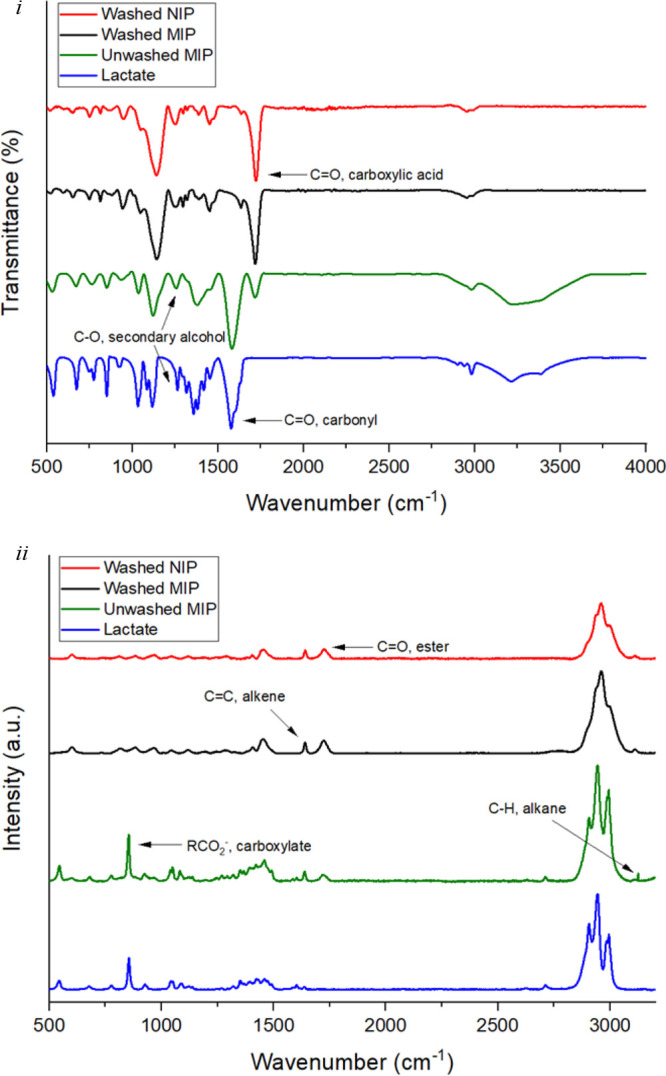
(i) FT-IR trace and (ii) Raman trace comparing the identifiable functional groups present within washed NIP, MIP, and unwashed MIP species with the addition of pristine lactate as a reference for the determination of the presence and/or absence of the template molecule.

FT-IR (Figure 2i) and Raman spectra (Figure 2ii) were obtained to identify the functional groups present within the polymerized 3D network and monitor washing effectiveness. The observed FT-IR peaks for the washed NIP and MIP structures are characteristic of the −C–O, C=O, and C=C bonds respresentative of the ester and alkene functional groups within EGDMA, and −C–O, −CH_3_, and C=C bonds significant of the carboxylic acid and alkene functional groups within MAA, respectively (Figure 2i).^39^ With regard to the unwashed MIP, there is a visible peak around 1580 cm^–1^ attributed to the carbonyl functional group (C=O bond), signifying the presence of lactate in this polymeric structure, which in the washed MIP is no longer visible, supporting effective washing protocols.^40^ Additionally, subsequent to washing protocols, there is a visible resolvement of the unified peak at around 1250 cm^–1^ observed in the unwashed MIP into multiple peaks as shown in both the washed MIP and NIP traces at around 1250–1320 cm^–1^, signifying the disappearance of the C–O bond within the secondary alcohol functional group of lactate.^40^ This is further reinforced by the disappearance of the broad −OH peak, which is observed between 3400 and 3200 cm^–1^ in the pristine lactate and unwashed MIP trace but not in the washed MIP trace, signifying successful lactate extraction from the washed MIP. Data collected from Raman spectroscopy further supports the FT-IR observations, whereby characteristic peaks attributed to the −CH_3_, C=O, and C–COO bonds present in the structure of EGDMA and C=O, CH_3_, and C–CH_3_ bonds present in the MAA structure, respectively, are visibly displayed (Figure 2ii).^41,42^ Furthermore, around 780 cm^–1^ the RCO_2_^–^ bond, attributed to the carboxylate functional group existing within the lactate structure, can be seen in both the unwashed MIP and pure lactate structures but is not detected in the washed MIP.^40,43^ The highlighted functional features in the FT-IR (Figure 2i) and Raman spectra (Figure 2ii) provide further characterization evidence of successful lactate–MIP preparation and effective target analyte extraction protocols (see Tables S2 and S3 for further data).

**Figure 3:**
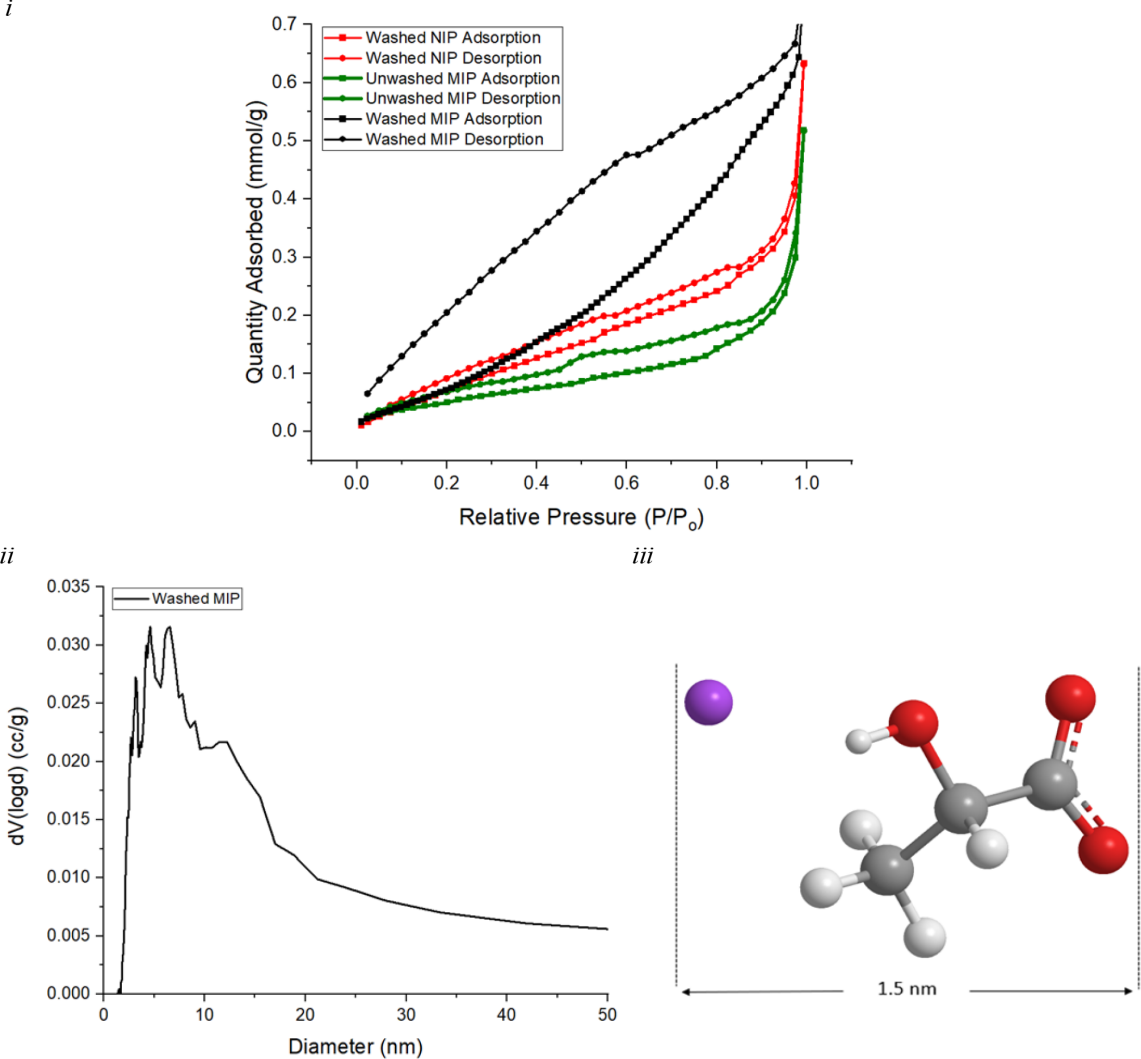
BET isotherms for (i) washed NIP, unwashed MIP, and washed MIP; (ii) pore size distribution of washed MIP powders calculated using Barrett–Joyner–Halenda analysis of the adsorption; (iii) estimated molecular diameter of a lactate molecule.

The porosity of the MIP and NIP powders were also investigated. A summary of the surface areas and pore size distributions is provided in Table 1. The calculated surface areas show a 1.3-fold increase for the MIP (ca. 17.6 m^2^/g), compared to NIP (ca. 14.0 m^2^/g), which is attributed to the increase in MIP porosity, a result of successfully installed lactate cavities. Subjecting the NIP to the template extraction phase (i.e., PBS washings) aims to maximize the comparison between the systems and assess the effects of nonspecific binding. It is assumed that mechanical milling does not influence the templated pore size (5–30 nm) as the pores are 1000-fold smaller than the milled MIP particle sizes (ca. 7 μm). Moreover, mechanical milling increases the surface area available for PBS penetration resulting in more efficient template removal.^44−46^ The isotherm obtained from nitrogen adsorption of the MIP particles shows Type IV shape, following the International Union of Pure and Applied Chemistry (IUPAC) characterization, suggestive of molecular adsorption in mesoporous adsorbents (Figure 3i).^47^ The hysteresis loop observed in the MIP isotherm occurs at the multilayer range of physisorption and is attributed to capillary condensation occurring in mesopores with increasing relative pressure.^48^ This feature takes the form of a H3-type hysteresis loop, which is indicative of undefined pores due to nonrigid structures or interconnectivity within a porous framework. This structure is reinforced by low pressure hysteresis, often associated with the deformation of nonrigid pore walls.^48^ Such features are absent from the unwashed MIP and a similar observation is noted for the corresponding NIP isotherm (Figure 3i). The mesoporous nature of the MIP particles was further investigated through calculation of the particle size distribution (PSD), the resulting plot (Figure 3ii) shows a broad PSD with diameters between 2 and 30 nm, which is within the range of mesoporous materials according to IUPAC nomenclature (2–50 nm).^25^ As the largest interatomic distance within lactate measures approximately 1.5 nm (Figure 3iii), interconnectivity of pores (e.g., clustering of cavities) is possible during complexation. Alternatively, binding sites formed by the imprinting process could be interconnected by an additional porous network, a result of porogen extraction. This analysis, together with the Type IV-H3 nature of the isotherm, suggests that it is possible that multiple cavities have merged to form larger ones, instead of individual smaller ones. Ultimately, the MIP powder demonstrates a larger specific surface area, suggestive of porosity installment via the imprinting process, and is indicative of a greater binding potential to lactate.

**Table 1:** MIP and NIP Surface Area Calculated Using Brunauer–Emmett–Teller Theory and Pore Size Distributions Calculated Using Barrett–Joyner–Halenda Analysis

	Mass (mg)	Surface area (m^2^/g)	Pore volume (cc/g)	Pore diameter Dv(d) (nm)
MIP	150	17.6	0.031	3.16
MIP^a^	152	7.84	0.019	2.13
NIP	155	14.0	0.025	2.26

a Indicates unwashed MIP.

### Binding Properties of MIP

To assess the degree of lactate imprinting in the polymeric network, MIP and NIP powders were exposed to PBS (0.1 M, pH 8.0), containing gradient concentrations of the target lactate from 0.1 to 1.7 mM demonstrating a LoD of 0.162 mM (see eq S1 in the Supporting Information), where this value is in line with the analytical performance of other lactate detection devices (see Table 4). In addition, the MIP imprinting factor (IF) efficiency was calculated using eq 1, where the equilibrium binding capacity (*Q*), i.e., the amount of analyte bound by the MIP and NIP particles (mol/g), denoted by *Q*_MIP_ and *Q*_NIP_, respectively, was first determined. *Q* (eq 2) describes the divergence between the initial amount of target analyte in the introduced solution mixture relative to the amount in the supernatant, where *C*_i_, *C*_f_, *m*, and *V* define the initial concentration of the target analyte in the introduced mixture, equilibrium concentration of the target analyte in the solution mixture, mass of the polymer, and volume of the analyte solution mixture, respectively.^49−52^ Recapturing experiments were conducted by separately submerging an equal mass of polymer powders (1 g) in a 1.7 mM of lactate solution and shaking vigorously, and the final concentration of lactate in the solution mixture was measured (λ = 206 nm) (see Figure S2 and the Experimental Section) and determined via the calibration curve (Figure S6i). The adsorption percentage and capacity of lactate captured by MIP and NIP species were calculated using eq 2, facilitating the analysis of the IF (eq 1). Finally, the specificity adsorption ratio (α), which describes the selectivity degree of the MIP species in relation to the NIP species, was calculated using eq 3.^53^

The concentration of the eluent collected from submerging MIP powders in a 1.7 mM lactate solution decreased by an average of 1.08 mM to a final average concentration of 0.62 mM (σ 0.0337, *n* = 3) evidencing that the MIP had captured an average of 63.5% (σ 1.91, *n* = 3) of the lactate solution introduced into the imprinted species. The eluent collected from the NIP powder decreased by an average of 0.16–1.54 mM. This ca. 10% retention of lactate is attributed to nonspecific adsorption. The experimental washing/rebinding protocol was repeated for each species (MIP and NIP), highlighting the reusability of these affordable materials. In addition, MIP and NIP powders from a different batch were assessed, highlighting the repeatability of the fabricated molecularly imprinted powders. These results are in agreement with another reported lactate-based MIP, where a MIP designed for the selective solid-phase extraction of phospholipids from human milk signified a reuse capability of up to 20 and 6 times in the case of phospholipids standards and human milk fat samples.^54^ The presented experimental values are summarized in Table 2.
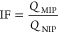
1
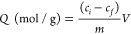
2
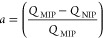
3

**Table 2:** Synthesized MIP and NIP Capture of a 1.7 mM Lactate Solution

Species	Eluent concentration after lactate solution saturation (mM)	Eluent concentration after PBS washing (mM)	Recapturing capacity (%)	IF	α
MIP	0.62 ± 0.02	0.32 ± 0.01	63.5 ± 1.91	6.86 ± 1.02	0.82 ± 0.022
NIP	1.54 ± 0.01	0.22 ± 0.03	9.34 ± 1.10		

The successful installment of lactate cavities within the MIP compared to the NIP was further supported by the immediate washing with PBS. New eluents were collected by submerging the same polymer powders in PBS, thus washing out rebound lactate molecules from the MIP. An average concentration of 0.32 mM (σ 0.011, *n* = 3) was recovered from the MIP powder on the second wash. Approximately half of the MIP-bound lactate was retrieved from the PBS washing, indicating a strong binding affinity of lactate molecules toward the MIP. One might consider that the MIP retention capacity (average of 63.5%) of the lactate solution could be further improved, perhaps via a different solvent choice. For instance, it is possible that within this polymeric system both lactate and MAA have strong interactions with that of methanol due to a high solvation value of the selected porogen. It is probable that the solvent choice is encouraging the formation of interactions between methanol and lactate, shielding the molecularly imprinted cavities, and weakening interactions with that of the functional monomer (MAA). Thus, this could reduce slightly the MIPs’ molecular recognition ability.^55^

In addition, as evidenced by the NIP species, nonspecific binding sites are present (ca. 10%). These sites can also be present within the MIP species, as a consequence of excess functional monomer, which is a common drawback of the noncovalent imprinting approach, often promoting the formation of a heterogeneous distribution of binding sites for prepolymerization complex stabilization.^56,57^ This result is in agreement with previous studies, whereby the structural parameters and sorption properties of MAA-EGDMA copolymers can drastically vary according to changes in the MAA to EGDMA ratio,^58−60^ including our previous work, where the number of cross-linker molecules relative to the template impacted the quality of imprinting and subsequent recapturing capacity.^25^ Nevertheless, the average IF for this MIP was calculated at 6.86 (σ 1.02, *n* = 3). Since successful imprinting is attributed to values greater than 1 (IF > 1), this MIP has pronounced lactate retention as a consequence of lactate cavity installment rather than nonspecific binding within the polymer matrix. Moreover, this MIP IF efficiency appears to have overperformed in comparison to other MIP species of comparable polymeric systems (e.g., MAA-EGDMA), where a greater IF efficiency is indicative of a stronger binding affinity.^61^ IF values of 6.45, 3.90, 3.53, and 2.80 for target analytes including cortisol, solanesol, gallic acid, and Congo red, respectively, have been reported.^25,52,62,63^

### Selectivity of Lactate MIP

With respect to α, values greater than 1 (α > 1) are indicative of an MIP’s capability to differentiate between structurally similar species.^64^ In this study, an average α value of 0.85 (σ 0.022, *n* = 3) has been demonstrated. This value is relatively close to 1 and therefore signifies this MIP’s potential to proficiently distinguish between the template analyte and those structures sharing a similar resemblance. However, it is likely there are modest limitations to this MIP’s ability to differentiate between structural interferants.

Lactate exists in a complex matrix of biomolecules. Thus, to quantify the selective capabilities of the system, MIP and NIP powders were submerged in a 1.7 mM concentration of malic acid and sodium 2-hydroxybutyrate. These molecules were selected due to their structural resemblance (e.g., functional groups) to that of lactate (Figure S1), facilitating similar hydrogen bonding. One would expect both these molecules to interact with MAA in a manner similar to that of lactate, sharing identical points of interaction. Therefore, a reduced malic acid and sodium 2-hydroxybutyrate capture by the MIP in comparison to lactate uptake would strongly indicate lactate selectivity. For consistency purposes, the concentration ranges for all target analytes (e.g., lactate, malic acid, and sodium 2-hydroxybutyrate) were kept the same.

To determine the affinity between malic acid and sodium 2-hydroxybutyrate molecules with that of lactate imprinted recognition, identical methods of rebinding (see Figure S2 and Experimental Section) were used when calculating the IF efficiency. The eluent concentration collected from submerging MIP powders in 1.7 mM of malic acid and sodium 2-hydroxybutyrate, respectively, decreased by 0.38 to 1.32 mM and 0.43 to 1.27 mM, evidencing that the MIP had captured 22.6 and 25.2%, respectively (Table 3). The eluent collected from submerging NIP powders in malic acid and sodium 2-hydroxybutyrate decreased by 0.16 to 1.54 mM and 0.18 to 1.52 mM, respectively. This ca. 10% retention of malic acid and sodium 2-hydroxybutyrate is attributed to nonspecific adsorption as expected given the lactate nonspecific adsorption values (Table 3).

**Table 3:** Synthesized MIP and NIP Capture of a 1.7 mM Malic Acid (MIP_MA_) and Sodium 2-Hydroxybutyrate (MIP_S2-HB_) Solution, Respectively

Species	Eluent concentration after lactate solution saturation (mM)	Eluent concentration after PBS washing (mM)	Recapturing capacity (%)
MIP_MA_	1.32	0.27	22.6
NIP_MA_	1.54	0.16	9.28
MIP_S2-HB_	1.27	0.29	25.2
NIP_S2-HB_	1.52	0.18	10.1

The data indicates that there are some interactive forces occurring between the interferents and the lactate installed cavities. These findings agree with the previously stated hypothesis where these interferents share a structural resemblance to that of lactate. Therefore, one would expect to see some binding interactions similar to that of lactate, particularly since MIPs tend to form high-affinity binding sites complementary in shape, size, and chemical functionality to that of the template in question and closely related analogues.^65^ Thus, a 22.6 and 25.2% retention of malic acid and sodium 2-hydroxybutyrate, respectively, by the lactate synthesized MIP corroborates that the main sites of interaction within this polymeric system are occurring between the carboxylic acid functional group of MAA and the carboxylate and alcohol functional groups of lactate in the form of noncovalent bonds (e.g., hydrogen bonding). Moreover, the retention of electron donors such as lactate, malic acid, and sodium 2-hydroxybutyrate strongly indicates that MAA (redox agent) is performing more like an electron acceptor than a donor, attributed to these binding interactions. Nonetheless, the retention of the interferents is significantly less than the retention observed for target lactate molecules 63.5% (σ 1.91, *n* = 3). This approximate 3-fold decrease in retention, detected for the interferrents compared to the lactate molecules, despite all molecules being exposed to identical chemical and environmental conditions throughout the rebinding process, signifies the interferent limitations toward MIP cavity saturation.

### Analytical Performance Comparison

In the further development and integration of these MIP powders into devices, the analytical performance has been compared to current lactate detection devices (not all utilizing MIP detection) as shown in Table 4. Regiart et al. designed an enzyme-based sensor with a wide linear range and high sensitivity toward the simultaneous detection of lactate and glucose.^8^ Booth et al. also developed a sensitive sensor for neurometabolic lactate monitoring, highlighting possible applications of fiber-based biosensors for direct monitoring of brain metabolites in the context of injury, where miniaturization is required.^10^ Although some of the devices report a wider linear range and lower sensitivities, their concentration levels are not in the specific range we are targeting that can indicate infection. Moussa and Zhou et al. focused their efforts on the continuous monitoring of lactate levels during periods of exercise and the detection of lactic acid as a cancer biomarker, respectively.^66,67^ Similarly, Xuan et al. fabricated a wearable lactate sensor for sweat analysis, demonstrating a linear range of response significantly below the anticipated lactate levels in sweat together as a promising technology for the sports domain.^9^

**Table 4:** Analytical Performance Comparison of Current Lactate Detection Devices

Sensor Development	Detection Method	Linear Range	LoD	Sample	Ref
Electrochemical	Amperometric	0–1000 μM	6.1 μM	Blood serum	(8)
Electrochemical	Amperometric	0–3 mM	19 μM	Brain and peripheral tissue	(10)
Electrochemical	Voltametric	10–250 μM	0.726 μM	Serum	(66)
Electrochemical	Amperometric	1–220 μM	0.003 μM	Sweat	(67)
Electrochemical	Amperometric	1–50 μM	0.11 mM	Sweat	(9)
OECT	Colorimetric	0–1 mM	1.9 mA/mM	Sweat	(68)
Electrochemical	Amperometric	0–24 mM	4.8 μA/mM^a^	Sweat	(69)
Chemical	HILIC-ELSD^b^	20–400 μg/mL	1.4, 2.6, 3.5 μg/mL^c^	Human milk	(54)
Chemical	UV–Vis	0.1–1.7 mM	0.162 mM.	PBS	This study

a Normalizes to 68 μA cm–2/mM when accounting for the surface area of the sensor.

b Hydrophilic interaction liquid chromatography in combination with evaporative light scattering detection.

c Reported for three different phosphocholines (phosphatidylethanolamine, phosphatidylcholine, and sphingomyelin).

Where the sample media constitutes sweat, our MIP powders demonstrate greater sensitivity, with a wider linear range than the organic electrochemical transistor (OECT) sensor. Despite the Currano group’s ability to develop an OECT as a noninvasive wearable sensor for monitoring lactate in sweat, this device’s sensing range was limited to concentrations below 1 mM.^68^ Furthermore, studies conducted by Payne et al. demonstrated the inability of enzymatic lactate sensors to give accurate numerical readings for continuous health monitoring due to their limitations in reacting selectively to the analyte of interest, owing to their unpredictability in changing environments.^69^

## Conclusion

In the development of MIPs as materials capable of specific lactate detection for future biodevice integration, this study has shown that the template–monomer assemblies successfully formed, and the spatial integrity of the imprinted species after lactate template extraction was conserved. Thermal characterizations confirmed interactions between the template–monomer complex within the MIP species while indicating that lactate could be incorporated within the polymer matrix without significant alteration of the material’s thermal properties. Surface area analysis supported the successful installation of lactate cavities by demonstrating a 1.3-fold increase with respect to the MIP (ca. 17.6 m^2^/g), compared to NIP (ca. 14.0 m^2^/g), while simultaneously highlighting the likelihood of interconnected networks and possible merging of cavities, which could be a limiting factor to this MIP’s binding capacity. Moreover, the experimental results from the rebinding of lactate molecules have confirmed the successful synthesis of a reusable, reliable, repeatable, and reproducible sensitive-lactate MIP, demonstrating a recapturing capacity of up to 63.5%. Additionally, the selective capabilities were observed via structurally similar analogues including malic acid and sodium 2-hydroxybutyrate, performing as interferents toward lactate installed cavities.

Additionally, the lactate-specific MIP has provided a detailed insight into the predominant hydrogen bonding occurring between the carboxylic acid functional group of MAA and the carboxylate and alcohol functional groups of lactate. Understanding these specific intermolecular mechanisms between the template analyte and structural interferents and the functional monomer enables experimentalists to make informed decisions regarding monomer-target and porogen selections, and their possible sites of interaction for improved molecular imprinting.

Moreover, performing the recapturing analysis in buffer solutions offers a potential insight into template-monomer interactions within biofluids (e.g., sweat, saliva, and interstitial fluid), which is encouraging for future minimally invasive diagnostic healthcare purposes (e.g., interstitial fluid MIP-based biosensors). This study offers a possible alternative to overcome limitations of using antibody/enzyme-based detection mechanisms in sensors by utilizing MIPs that could support self-health monitoring and management at the point-of-care.

## Experimental Section

### Materials and Methods

Sodium lactate (lactate), 2,2′-azobis(2-methylpropionitrile) (AIBN), methacrylic acid (MAA), ethylene glycol dimethacrylate (EGDMA), phosphate-buffered saline (PBS) tablets (pH 8.0 ± 0.02, 0.1 M), anhydrous dichloromethane >99.8% purity containing 40–150 ppm amylene as a stabilizer, and anhydrous methanol of 99.8% purity were all obtained from Sigma-Aldrich and used in the synthesis of both MIP and NIP powders. All chemicals were used as received with the exception of MAA and EGDMA, which had inhibitors removed by gravity filtration via prepacked aluminum oxide (activated, basic) columns designed for the removal of hydroquinone and monomethyl ether hydroquinone. Deionized (DI) water and ultrapure water (resistivity of 18 MΩ cm^–1^) were produced using a Purelab Chorus 1 Complete water purification system (Elga LabWater, Veolia Water Systems Ltd.).

Polymer powders were synthesized by first decanting aluminum oxide into two syringes filled with glass fibers. MAA as a functional monomer and EGDMA as a cross-linker were filtered (separately) through the columns under gravity. Once the inhibitors were removed, MAA (0.24 mL, 2.4 mmol) and EGDMA (2.26 mL, 12.0 mmol) were combined in a reaction vessel with the addition of 4 mL of dichloromethane. Lactate (44.8 mg, 0.4 mmol) as the template molecule was then added and solubilized with the addition of 0.4 mL of anhydrous methanol. After the reaction vessel was concealed from light, AIBN (16.4 mg, 0.1 mmol) was added as the initiator. The mixture was degassed with nitrogen for 25 min and then photochemically polymerized for 30 h using a standard laboratory UV reactor (Cole-Parmer 4-W UV lamp) at a wavelength of 365 nm and kept at 4 °C using a recirculatory bath (Isotemp). The obtained colorless and translucent polymer was manually ground using a pestle and mortar, mechanically milled (Retsch MM 400) at a frequency of 25 Hz for 25 min, and sieved using a 0.25 mm mesh.

### Selection of Functional Monomer, Cross-Linker, and Solvents

The rationale for this specific MAA-EGDMA-AIBN system where MAA, EGDMA, and AIBN were selected as the functional monomer, cross-linker, and photoinitiator, respectively, has been discussed in detail elsewhere.^25^ However, in brief, the presence of the carboxylic functional group in MAA enables hydrogen bonding to occur in a manner similar to biological recognition systems.^70^ EGDMA encourages the conservation of spatial integrity subsequent to lactate extraction, and AIBN is a common radical initiator often adopted in bulk polymerization syntheses.^71^ Polymerization was carried out at 4 °C to promote MIP recapturing capacity and selectivity by decreasing the kinetic energy and simultaneously increasing the stability of the prepolymerization complex.^72−74^ Anhydrous dichloromethane was the solvent of choice as its nonpolar nature promotes stability during polymerization, avoiding any reactions with free radicals that could interfere with template–monomer interactions. To encourage the solubility of lactate, 10% (volume) anhydrous methanol was added as it is a protic solvent capable of hydrogen bonding.^75^

### Synthesis of Lactate MIP

All imprinted species were synthesized via bulk polymerization, whereby the self-assembly approach was utilized due to its ability to promote noncovalent forces like genetic recognition systems.^71^ Mechanical milling was utilized to achieve polymer powder particles of uniform size and shape. In addition, this technique increases the surface area of MIP particles, facilitating better PBS penetration, resulting in more successful template extraction (Figure S3i) and greater lactate recapture.^45,46^ A control nonimprinted polymer (NIP) was prepared under identical conditions, excluding lactate. The described method outlines the synthesis of a lactate-specific MIP in a 1:6:30:0.25 ratio of lactate, MAA, EGDMA, and AIBN, respectively. It is important to note that preventative measures were taken to avoid degradation of lactate, malic acid, and sodium 2-hydroxybutyrate in solution for those mixtures that required longer storage life (ca. 2 weeks). These solutions were stored between 2 and 8 °C, where necessary.

### Extraction of Template Molecules

Lactate molecules were extracted by successive washings with PBS, a biocompatible aqueous fluid, serving as a pseudobiological fluid and providing insight into potential biotic mechanisms. Polymer powders were suspended in a PBS solution, well-mixed using a rotating mixer (Fisherbrand) for 15 min, and centrifuged (Medifuge) for 10 min at 4500 rpm. The products were dried in a heated cabinet at 30 °C for 48 h. The decreasing levels of lactate was followed spectrophotometrically (λ = 206 nm) using a Jenway 7205 spectrophotometer (Figure S3i). Absorbance was measured against a background of PBS until the template could no longer be detected. For the purposes of complete comparative analysis, both the MIP and NIP were exposed to consistent chemical conditions, including washing steps (Figure S3i,ii, where Figure S3iii displays a neat trace of lactate as a reference) throughout their syntheses. Moreover, evidence has suggested that skipping the template extraction protocol for the NIP could lead to unrealistic selectivity factors compared to that of the MIP.^49,76^

A lactate calibration curve (*R*^2^ = 0.9949) measuring in the linear range of 0.1–1.7 mM (Figure S6i) was constructed via UV–Vis absorbance bands measured at λ = 206 nm against a background of PBS. Prior to the selection of PBS, methanol and ethanol were tested for their solubilizing capability of the template molecule in the prepolymerization mixture (Figure S7i,ii). Both solvents were unsuccessful in quantitatively measuring the extraction of lactate from the MIP as it was difficult to identify a specific wavelength for lactate detection in the presented solvents and consistency in wavelength intensity. Malic acid and sodium 2-hydroxybutyrate stock solutions with concentrations of 0.1, 0.2, 0.3, 0.5, 0.8, 1.1, 1.4, and 1.7 mM, respectively were freshly prepared by the previously described method. For continuity, the concentration range (0.1–1.7 mM) was kept the same as lactate. Initially, increments of 0.3 mM were selected, starting from 0.5 to 1.7 mM to determine the overall relationship, if any, between absorbance and concentration within this system. To further investigate the observed trend, smaller increments of 0.1 mM starting from 0.1 to 0.3 mM were measured to gain a deeper insight into the trend and to identify both the reliability and sensitivity of the developed standard curve. Malic acid and sodium 2-hydroxybutyrate absorbance bands λ = 206 nm were measured against a background of PBS, and these results were plotted to formulate a calibration curve (*R*^2^ = 0.9917 and 0.9961, respectively) (Figure S6ii,iii).

### Recapturing of Lactate and Selectivity of Malic Acid and Sodium 2-Hydroxybutyrate

1 g of MIP and 1 g of NIP were submerged (separately) in lactate solutions (1.7 mM, 10 mL) in a sealed borosilicate glass vial placed on a laboratory shaker (Orbit Orbital Shaker) for 60 min at 60 rpm (Figure S2i). Polymer powders and eluents were separated by centrifuging (Medifuge) three times for a period of 15 min at 4500 rpm (Figure S2ii). Collected eluents were syringed through 22 μm PTFE filter membranes twice. This procedure was directly repeated using 10 mL of PBS to wash out captured lactate molecules within the imprinted polymer powders. For selectivity determination, the described procedure was repeated for MIP and NIP powders (1 g) submerged (separately) in malic acid and sodium 2-hydroxybutyrate solutions (1.7 mM, 10 mL). All collected eluent samples were analyzed via UV–vis spectroscopy (Figure S2iii).

### Structural Evaluation of Polymers

#### Thermal Characterization of Polymers

The glass transition temperature (*T*_g_) of MIPs and NIPs were analyzed by differential scanning calorimetry (DSC) using a Thermal Advantage DSC Q20 machine from TA Instruments controlled by the Q Series program and equipped with a Thermal Advantage Cooling system 90. DSC operated under a nitrogen purge with a flow rate of 18 cm^3^/min. Samples were loaded into 10 μL *T*_zero_ aluminum pans and heated or cooled at a rate of 10 °C/min. The heat flow was recorded against an empty 10 μL *T*_zero_ reference pan. The onset of thermal degradation, *T*_d_, was recorded by thermogravimetric analysis (TGA) using a Setsys Evolution TGA from Setaram. The Calisto program was used to collect and process data. Samples were loaded into a 170 μL alumina crucible and heated from 25 to 350 °C at a rate of 10 °C/min under a flow of argon.

#### Spectroscopy

Fourier transform infrared (FT-IR) spectroscopy was performed as a structural analysis via functional group determination using a PerkinElmer Frontier Opticia FT-IR microscope using the program software Spectrum. Raman spectroscopy was conducted using a Renishaw InVia system with a 532 nm green laser set at a power of 69 mW and a 10 s exposure with three accumulations for point spectra.

#### Nitrogen Adsorption Isotherms and BET Surface Area

Nitrogen adsorption isotherms were performed on a 3Flex machine from Micromeritics, and calculations were performed within the MicroActive program. Samples (ca. 150 mg of NIP and MIP powder, respectively) were loaded into 9 mm wide glass tubes and degassed *in situ* with the aid of a heating chamber set at 100 °C. Adsorption of nitrogen was conducted at 77 K. From the collected adsorption data, the samples’ surface area was estimated using the Brunauer–Emmett–Teller theory and the samples’ pore size distribution calculated using Barrett–Joyner–Halenda analysis.

#### Microscopy

Polymer powders were examined under a JSM-6301F field emission scanning electron microscopy (FE-SEM). Before analysis, the samples were mounted onto aluminum stubs and evacuated overnight. The samples were then coated with a 20 nm layer of chromium using a Quorum Q150T S sputter coater. Samples were viewed at a working distance between 8 and 10 mm under an accelerating voltage of 5 kV (Figure S8i,ii). The average particle size and distribution of MIP and NIP particles were determined using optical microscopy. Each sample was viewed at a magnification of 10× using brightfield illumination on an Olympus BX51 microscope. Image captures (Figure S9i,ii) were taken using the cellSens Standard program and were analyzed within ImageJ: a scale of 160 pixels:100 μm was set. The threshold was adjusted to highlight particles of interest, a sample area was set, and particles within this area were analyzed in terms of their Ferret diameters. This process was repeated for a variety of different sample areas, and the mean values are reported in Table S4.

### Binding Evaluation of Polymers

#### Spectroscopy

Ultraviolet–visible spectroscopy was carried out using a Jenway 7205 spectrophotometer. Solutions were analyzed using a Suprasil quartz cuvette of spectral range 200–2500 nm, path length 10 mm, chamber volume 700 μL.

## Supporting Information Available

Chemical structure of malic acid and sodium 2-hydroxybutyrate; schematic of the general protocol for MIP capture of the target species; UV–Vis traces of MIP and NIP successive washings (template extraction using PBS), and neat spectra of template and interfering species; thermal polymer characterization (DSC and TGA traces); FT-IR and Raman chemical shifts; LoD calculation; lactate, malic acid, and sodium 2-hydroxybutyrate calibration curves; UV–Vis analysis of unsuccessful template extraction using methanol and ethanol; microscope images (FE-SEM and optical microscopy), and particle size distribution (PDF)
