# Supplementary material for: Fabrication of a Lactate-Specific Molecularly Imprinted Polymer toward Disease Detection
Source: ACS Omega. 2023 Feb 21;8(9):8732–42. doi: 10.1021/acsomega.2c08127 (PMC9996612; doi:10.1021/acsomega.2c08127)
Supplement: Supplementary file 1 — ao2c08127_si_001.pdf [file ao2c08127_si_001.pdf]

Fabrication of a Lactate-Specific Molecularly Imprinted Polymer Towards Disease Detection

Yasemin L. Mustafa [a, b] and Hannah S. Leese [a, b]*

[a] Materials for Health Lab, Department of Chemical Engineering, University of Bath, Bath, BA2 7AY, UK.

[b] Centre for Biosensors, Bioelectronics and Biodevices, University of Bath, Bath, BA2 7AY, UK.

***Corresponding author**

h.s.leese@bath.ac.uk

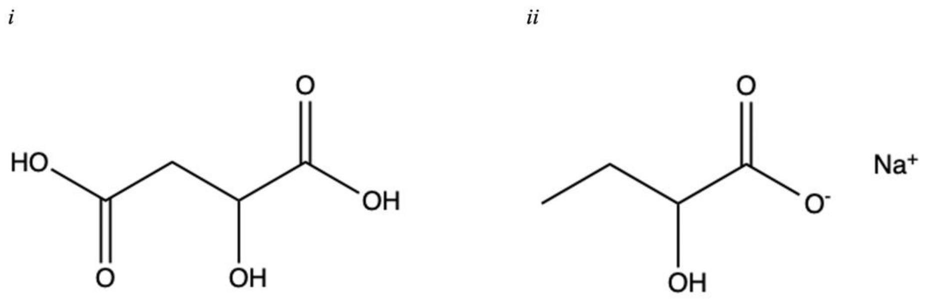

Figure S1. Structures of (*i*) malic acid and (*ii*) sodium 2-hydroxybutyrate.

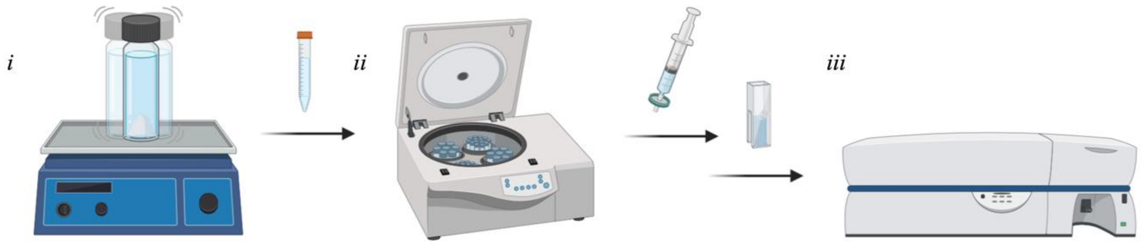

Figure S2. Schematic of the general protocol for MIP capture of the target species. Procedure involves (*i*) submerging MIP powders in a known concentration; (*ii*) collecting eluents subject to methods of separation, including centrifugation and filtration; (*iii*) analysing via UV-Vis spectroscopy.

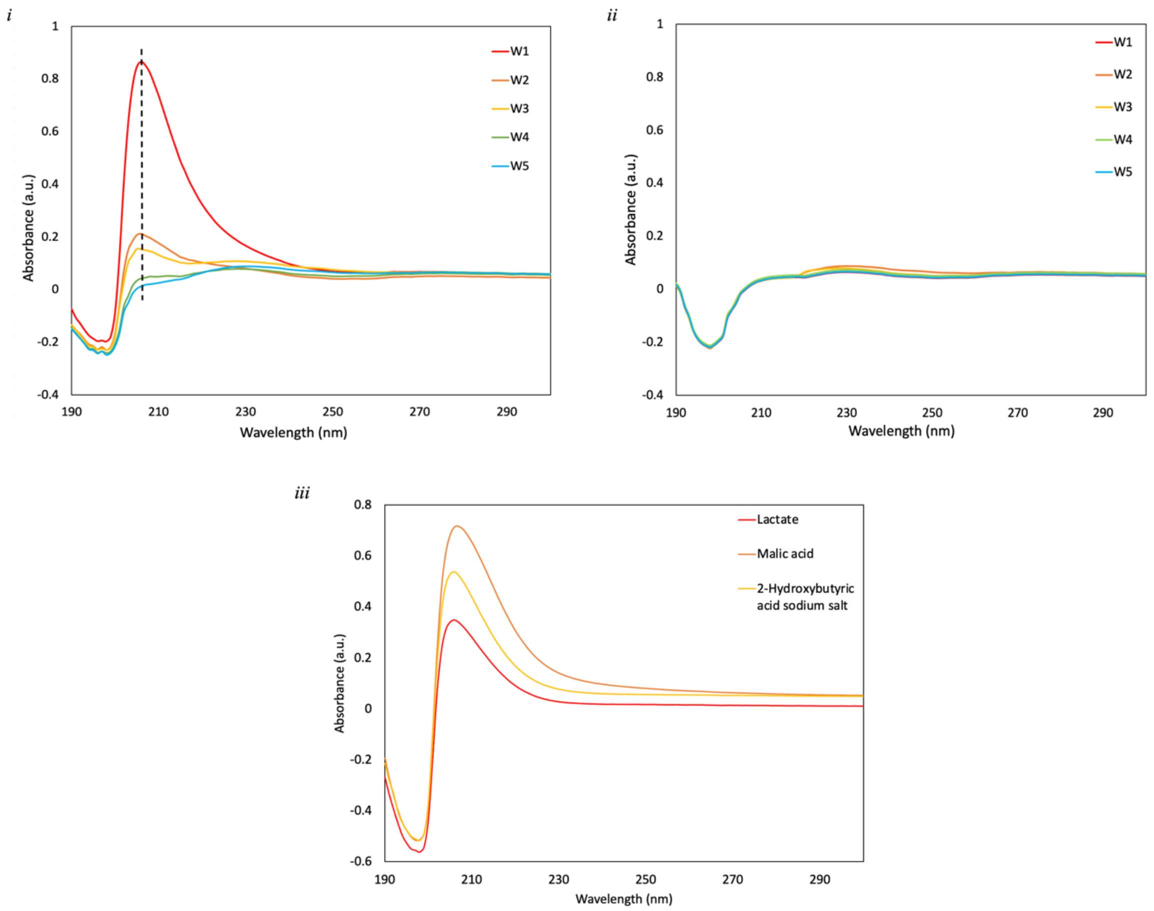

Figure S3. UV/Vis analysis of successive washings with PBS for (*i*) MIP, (*ii*) NIP, and (*ii*) neat spectra of lactate, malic acid, and sodium 2-hydroxybutyrate at 1.7 mM, respectively. The specific absorption of lactate at 206 nm has been highlighted.

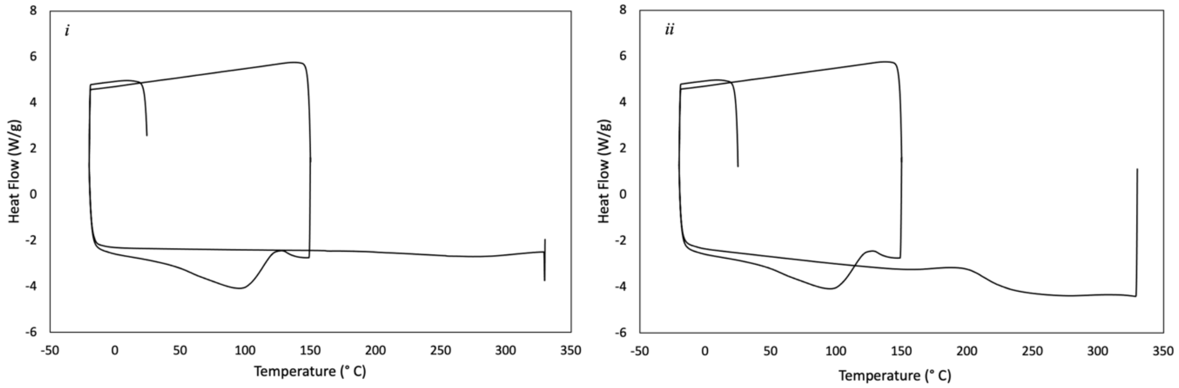

Figure S4. DSC trace of washed (*i*) MIP and (*ii*) NIP powders: first heating cycle ran from 25 to 150 °C under nitrogen at a rate of 10 °C/min. Second heating cycle ran from -20 to 330 °C at a rate of 10 °C/min, before cooling to 25 °C.

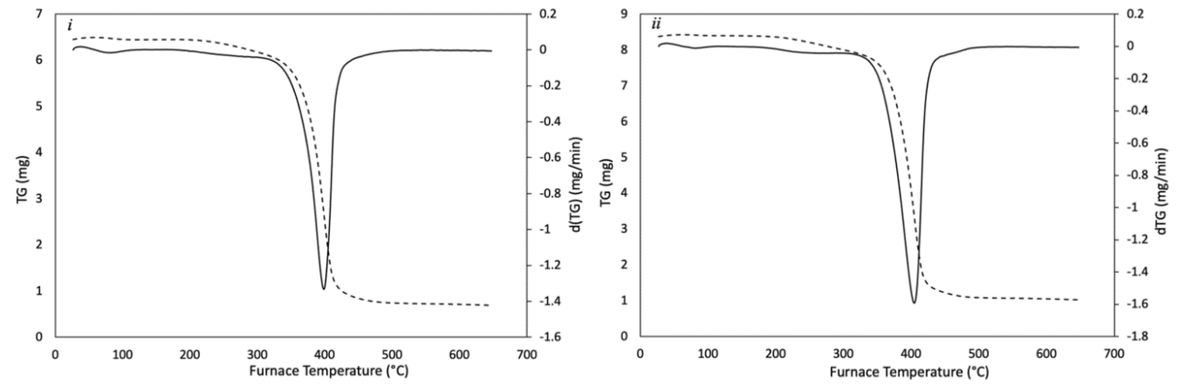

Figure S5. TGA traces of (*i*) washed MIP and (*ii*) NIP.

Table S1. Thermal decomposition and glass transition temperature of washed MIP and NIP species.

Species	Thermal decomposition (T_d)/ °C	Glass transition temperature (T_g)/ °C
MIP	219	200
NIP	224	204

Table S2. FT-IR frequencies and their correlating functional groups for EGDMA and MAA, respectively.

Ethylene glycol dimethacrylate (EGDMA)			
Wavenumber (cm^{-1})	Bond	Functional Group	Ref.
1630	C=C	Alkene	[1,2]
650			
1390	=C-H		
1450	C-H	Alkane	[1,2]
1320			
1290	C-O-C	Ester	[1,2]
1140			
Methacrylic acid (MAA)			
2954	C-H	Alkane	[2,3]
1720	C=O	Carboxylic acid	[2,3]
1030	O-H		
Sodium lactate			
1250	C-O	Secondary alcohol	[4]
1580	C=O	Carbonyl	[4]

Table S3. Raman frequencies and their correlating functional groups for EGDMA and MAA, respectively.

Ethylene glycol dimethacrylate (EGDMA)			
Wavenumber (cm^{-1})	Bond	Functional Group	Ref.
3109	C-H	Alkane	[1]
2959	$-CH_2$		
1724	C=O	Ester	[1]
Methacrylic acid			
1639	C=C	Alkene	[3]
1406	$-CH_3$	Alkane	[3]
1040	-C-C		
966			
Sodium lactate			
780	RCO_2^-	Carboxylate	[4,5]

The LoD was calculated based on the standard deviation of the response (Sy) of the curve and the slope of the calibration curve (S) at levels approximating the LoD according to Equation A. The standard deviation of the response has been determined via the standard deviation of y-intercepts of regression lines.

$$LoD = 3.3\left(\frac{S_y}{S}\right) \quad \textbf{(S1)}$$

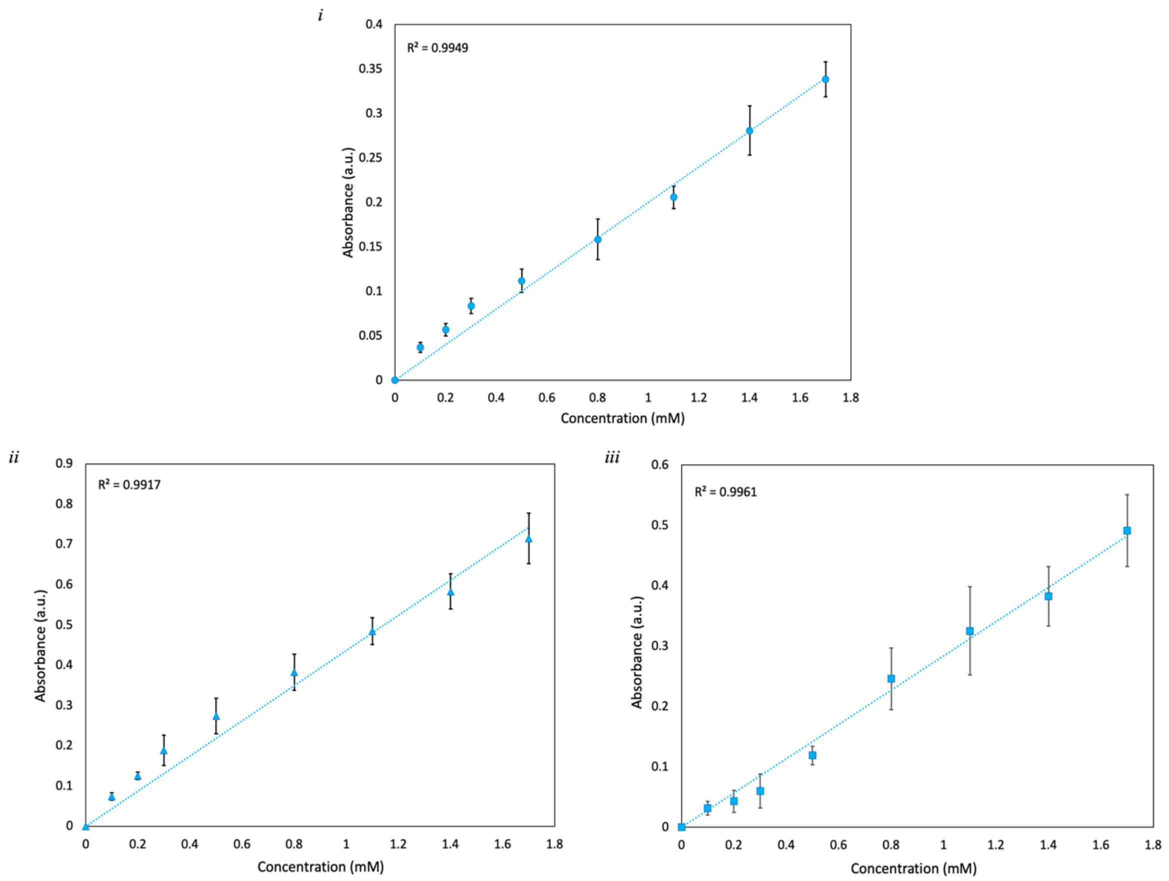

Figure S6. Calibration curve for (*i*) lactate ((l = 206 nm), (*ii*) malic acid (l = 206 nm), and (*iii*) 2-hydroxybutyric acid sodium salt (l = 206 nm) at concentrations of 0.1, 0.2, 0.3, 0.5, 0.8, 1.1, 1.4, and 1.7 mM, respectively.

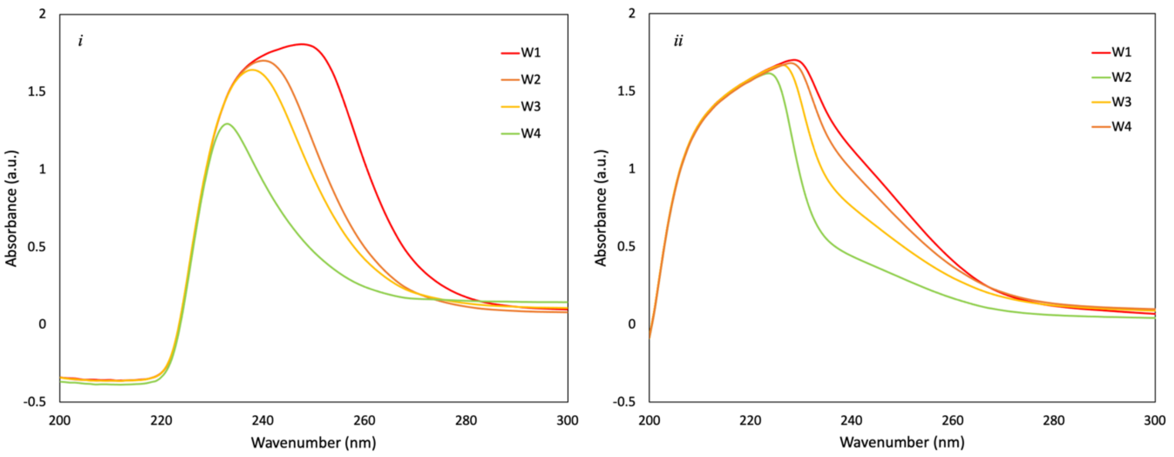

Figure S7. UV/Vis analysis of successive washings with (*i*) methanol and (*ii*) ethanol, highlighting the inability to successfully measure the removal of lactate molecules using the selected solvents.

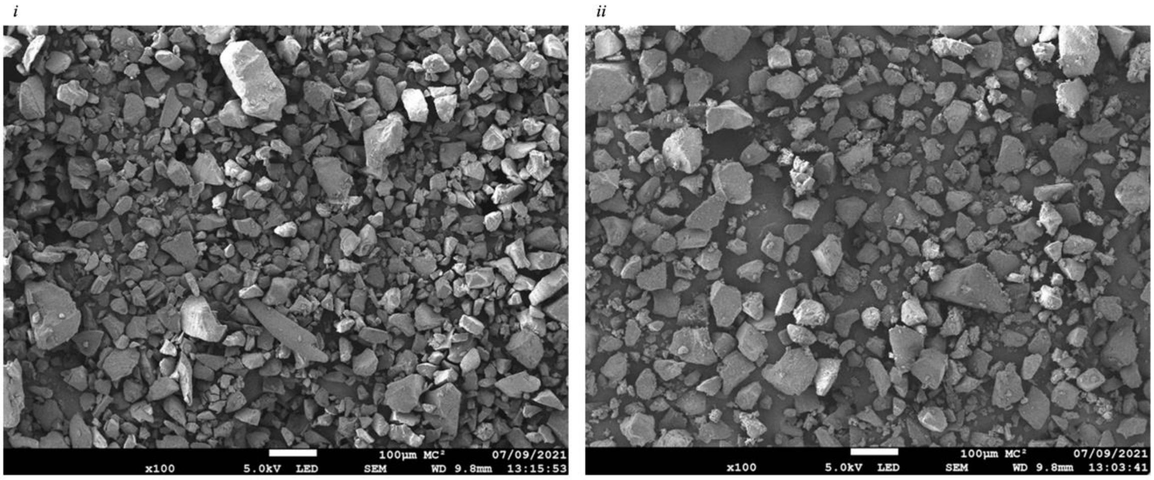

Figure S8. FE-SEM images of (*i*) washed MIP and corresponding (*ii*) NIP particles.

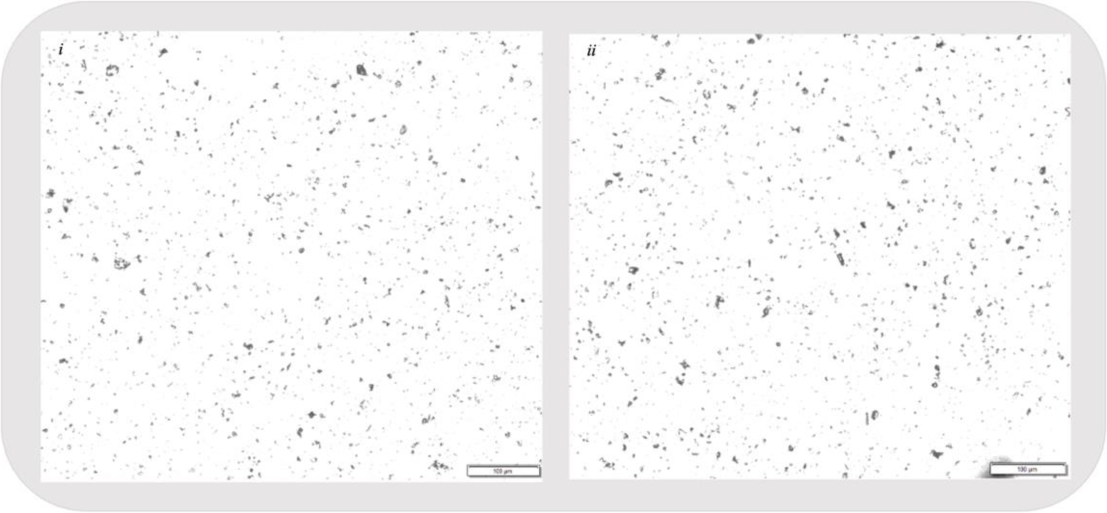

Figure S9. Optical microscope images of milled and washed (*i*) MIP and (*ii*) NIP particles used in particle size determination.

Table S4. Particle size distribution of three different washed MIP and NIP powder samples.

Samples	MIP Average Particle Sizes (μm)	NIP Average Particle Sizes (μm)
1	6.10 ± 1.15 (n = 21)	6.93 ± 1.22 (n = 21)
2	6.90 ± 1.32 (n = 21)	7.00 ± 1.40 (n = 21)
3	7.18 ± 1.19 (n = 20)	6.93 ± 1.22 (n = 20)
Total	6.72 ± 1.29 (n = 62)	6.95 ± 1.26 (n = 62)

References

(1) Chemicalbook, CAS Database - Ethylene Glycol Dimethacrylate. https://www.chemicalbook.com/ChemicalProductProperty_EN_CB8146599.htm (accessed 2021-09-12).

(2) Gomes, C.; Sadoyan, G.; Dias, R.; Costa, M. R. P. F. N. Development of Molecularly Imprinted Polymers to Target Polyphenols Present in Plant Extracts. *Processes* **2017**, *5* (4), 72.

(3) Chemicalbook, CAS Database - Methacrylic Acid. https://www.chemicalbook.com/ChemicalProductProperty_EN_CB9240023.htm (accessed 2021-09-12).

(4) Chemicalbook, CAS Database, Sodium Lactate. https://www.chemicalbook.com/ChemicalProductProperty_EN_CB6114077.htm (accessed 2021-10-27).

(5) Frost, R. L.; Kloprogge, J. T. Raman Spectroscopy of the Acetates of Sodium, Potassium and Magnesium at Liquid Nitrogen Temperature. *J. Mol. Struct.* **2000**, *526* (1–3), 131–141.
